# Association Between Peripheral Inflammation Biomarkers and Clinical Phenotypes in Patients With Medication Overuse Headache: A Cross‐sectional Study

**DOI:** 10.1002/brb3.71092

**Published:** 2025-11-21

**Authors:** Changling Li, Peiqi He, Mengmeng Ma, Yanbo Li, Jinghuan Fang, Qian Liu, Yang Zhang, Xin Jiang, Shiqin Li, Hui Lang, Ning Chen, Li He

**Affiliations:** ^1^ Department of Neurology West China Hospital of Sichuan University Chengdu Sichuan China

**Keywords:** AISI, medication overuse headache, peripheral inflammatory markers, SII, SIRI

## Abstract

**Background:**

The role of peripheral inflammation in medication overuse headache (MOH) remains inadequately understood. This study aimed to investigate biomarkers of peripheral inflammation in MOH patients and examine their relationship with clinical features.

**Methods:**

The study comprised 128 MOH patients and 132 age‐ and sex‐matched controls, including 60 episodic migraine patients and 72 healthy controls (HC). Inflammatory markers derived from blood cell counts were assessed in all participants. Clinical features, including headache days and intensity per month and days per month with acute medication, were documented.

**Results:**

In MOH patients, the aggregate index of systemic inflammation (AISI) was higher compared to healthy controls, and both MOH and episodic migraine patients had elevated systemic inflammation response index (SIRI) levels. AISI and SIRI were positively correlated with the days per month with acute medication, while systemic inflammation index (SII), SIRI, AISI, and other inflammatory markers correlated with headache duration. AISI proved more effective than SII and SIRI in diagnosing MOH. Furthermore, elevated SII and AISI levels were significantly associated with MOH after adjusting for age and sex.

**Conclusion:**

The study found significant correlations between SII, SIRI, and AISI and clinical phenotypes in MOH. Elevated SII and AISI levels were significantly linked to MOH, implying that these peripheral inflammation biomarkers could enhance the understanding of MOH.

## Graphical abstract

Systemic inflammation indices (SII, SIRI, and AISI) were linked to clinical phenotypes in medication overuse headache (MOH). Specifically, higher SII and AISI showed independent associations with MOH.

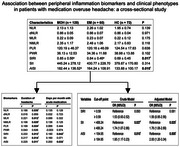

**Abbreviations**
AISI: aggregate index of systemic inflammation
ANOVA: One‐way analysis of variance
AUC: area under the ROC curve
dNLR: derived neutrophil‐to‐lymphocyte ratio
EM: episodic migraine
HC: healthy controls
ICHD‐3: International Classification of Headache Disorders, third edition
lt‐MOH: long‐term medication overuse headache
MLR: monocyte‐to‐lymphocyte ratio
MOH: medication overuse headache
NLR: Neutrophil‐to‐lymphocyte ratio
NMLR: the ratio of (neutrophil count + monocyte count)/lymphocyte count
NSAIDs: Non‐steroidal anti‐inflammatory drugs
PLR: platelet‐to‐lymphocyte ratio
PWR: platelet‐to‐WBC ratio
ROC: receiver operating characteristic curve
SII: systemic inflammatory index
SIRI: systemic inflammation response index
st‐MOH: short‐term medication overuse headache
TTH: tension‐type headache
WBC: white blood cells

## 1 Introduction

Medication overuse headache (MOH) is classified as a secondary chronic headache affecting approximately 60 million individuals globally, mainly due to excessive use of acute medications by those with migraine or tension‐type headache (TTH) (Ashina et al. 2023; Gosalia et al. 2024). MOH significantly impacts quality of life and raises healthcare costs, ranking eighteenth in global disability causes in 2013 (Aramruang et al. 2024; Ashina et al. 2023). The annual cost per MOH patient is €3561, nearly three times the expense for episodic migraine (EM) and ten times the cost for TTH (Linde et al. 2012). Despite its prevalence, awareness among clinicians and the public is limited (Green 2021; Lai et al. 2014), and MOH remains challenging to manage with no consensus on treatment (Gosalia et al. 2024). Research into pathophysiological mechanisms like inflammation is crucial for identifying potential therapeutic targets.

Recent studies indicate that neuroinflammation may be linked to the onset and/or progression of MOH (Gong et al. 2023; Wang et al. 2023), often occurring alongside chronic peripheral inflammation (Passaro et al. 2021; Vázquez‐Mojena et al. 2023). This connection can be assessed through blood inflammatory biomarkers, which offer an indirect, less invasive, and more practical approach to evaluating brain inflammation (Gaetani et al. 2020). MOH patients showed higher peripheral lymphocyte counts than migraineurs, suggesting chronic inflammation (Forcelini et al. 2011). Low‐grade systemic inflammation is a feature of MOH (Vuralli et al. 2024; Vuralli, Ceren Akgor, Gok Dagidir, et al. 2024). The neutrophil‐to‐lymphocyte ratio (NLR), monocyte‐to‐lymphocyte ratio (MLR), and platelet‐to‐lymphocyte ratio (PLR) have been proposed as novel biomarkers for assessing systemic inflammation in various headache disorders (Kömürcü et al. 2025; Lee et al. 2022). EM patients exhibited higher levels of NLR, PLR, and MLR compared to healthy controls (HC) (Yazar et al. 2020). MOH patients demonstrated a higher NLR than EM patients and HC, indicating a stronger immune response. A lower NLR was correlated with fewer headache days/month in MOH patients (Carlsen et al. 2023). There is a lack of research on MLR and PLR in MOH patients.

Biomarkers like systemic immunoinflammatory index (SII), systemic inflammation response index (SIRI), and aggregate index of systemic inflammation (AISI), derived from blood cell counts, better reflect inflammation and immune status than NLR and PLR (Cheng et al. 2023; Hamad et al. 2022; Hu et al. 2014; Urbanowicz et al. 2022). MOH patients also have higher systemic pro‐inflammatory cytokines, such as IL‐6, compared to EM patients and HC (Vuralli et al. 2024; Vuralli, Ceren Akgor, Gok Dagidir, et al. 2024). Despite cytokine analysis efforts, inflammatory indices like SII, SIRI, and AISI, which are cost‐effective and easy to measure, have not been studied in MOH.

Therefore, this study aimed to examine changes in inflammatory biomarkers from blood counts in MOH patients, EM patients, and HC, and to explore their relationship with MOH clinical phenotypes like headache frequency and analgesic use.

## 2 Methods

### 2.1 Participants

MOH patients were consecutively recruited from the tertiary headache clinic at West China Hospital between January 2021 and January 2024, meeting criteria of being aged 18–65, diagnosed with MOH according to the International Classification of Headache Disorders, third edition (ICHD‐3) criteria (IHS. 2018), having a history of migraine or TTH, and not using prophylactic medications for three months prior. The EM and HC groups were age‐ and sex‐matched to MOH patients. EM‐controls met ICHD‐3 criteria for migraine without aura (IHS. 2018) and had not used prophylactic migraine medications in the past three months. HC‐controls were included if they had no history of headaches. Participants were excluded if they: (1) were under 18 or over 65; (2) lacked data on peripheral inflammation biomarkers; or (3) had acute/recent infections, other chronic diseases, significant neurological or major psychiatric disorders, or systemic diseases. After a clinical assessment, two headache specialists (LC and HP) enrolled all participants.

### 2.2 Data Collection

Demographic data, including age and sex, were collected post‐enrollment. MOH and EM patients completed a questionnaire about their detailed headache history (such as duration of headache, any preexisting headache conditions, and duration of medication overuse), acute medication use, headache frequency and intensity (using the Visual Analogue Scale), and medication days over the past three months. For repeat clinic visitors, data from their first visit were used.

Blood samples were collected from MOH patients during headache‐free periods and from EM patients during interictal periods (headache‐free for three days before and after collection, without acute headache medications). All participants fasted for 8 to 10 hours before sampling at the outpatient clinic of West China Hospital. Blood indices, including erythrocyte, platelet, white blood cell (WBC), and leukocyte subsets (monocytes, lymphocytes, and neutrophils) counts, were measured using an XN‐9100 automatic blood cell analyzer (Hysen Micon Medical Electronics, Shanghai). These indices were reported in units of ×10^9^ cells/L. The calculated blood count‐derived inflammatory biomarkers were as follows (Cheng et al. 2023; Tiucă et al. 2023; Vázquez‐Mojena et al. 2023): NLR, derived neutrophil to lymphocyte ratio (dNLR), MLR, the ratio of (neutrophil count + monocyte count)/lymphocyte count (NMLR), PLR, platelet‐to‐WBC ratio (PWR), SIRI (neutrophil count × monocyte count/lymphocyte count), SII (platelet count × neutrophil count/lymphocyte count), and AISI (platelet count × neutrophil count × monocyte count/lymphocyte count).

### 2.3 Statistical Analysis

Statistical analyses were conducted utilizing Statistical Product and Service Solutions Software, version 22.0 (IBM, Armonk, USA) for Windows. Continuous variables were expressed as means ± standard deviation, while categorical variables were presented as absolute counts with corresponding proportions. Group differences among these variables were evaluated using one‐way analysis of variance (ANOVA), the Kruskal–Wallis *H* test, the Mann–Whitney *U* test, the chi‐square test, and Fisher's exact test, as appropriate. The Bonferroni correction was applied for multiple comparisons. Spearman rank correlation examined the link between inflammatory biomarkers and clinical characteristics in MOH patients. Receiver operating characteristic (ROC) curve analysis was used to determine the optimal cut‐off values for SII, SIRI, and AISI using the Youden index (sensitivity + specificity − 1), with the model's predictive accuracy assessed by the area under the ROC curve (AUC). The study examined the relationships between SII, AISI, and SIRI with MOH using both univariate and multivariate logistic regression analyses, accounting for age and sex. All analyses were performed using a two‐tailed approach, with a significance level set at *p* < 0.05.

## 3 Results

### 3.1 Baseline Characteristics and Inflammatory Indices

**FIGURE 1:**
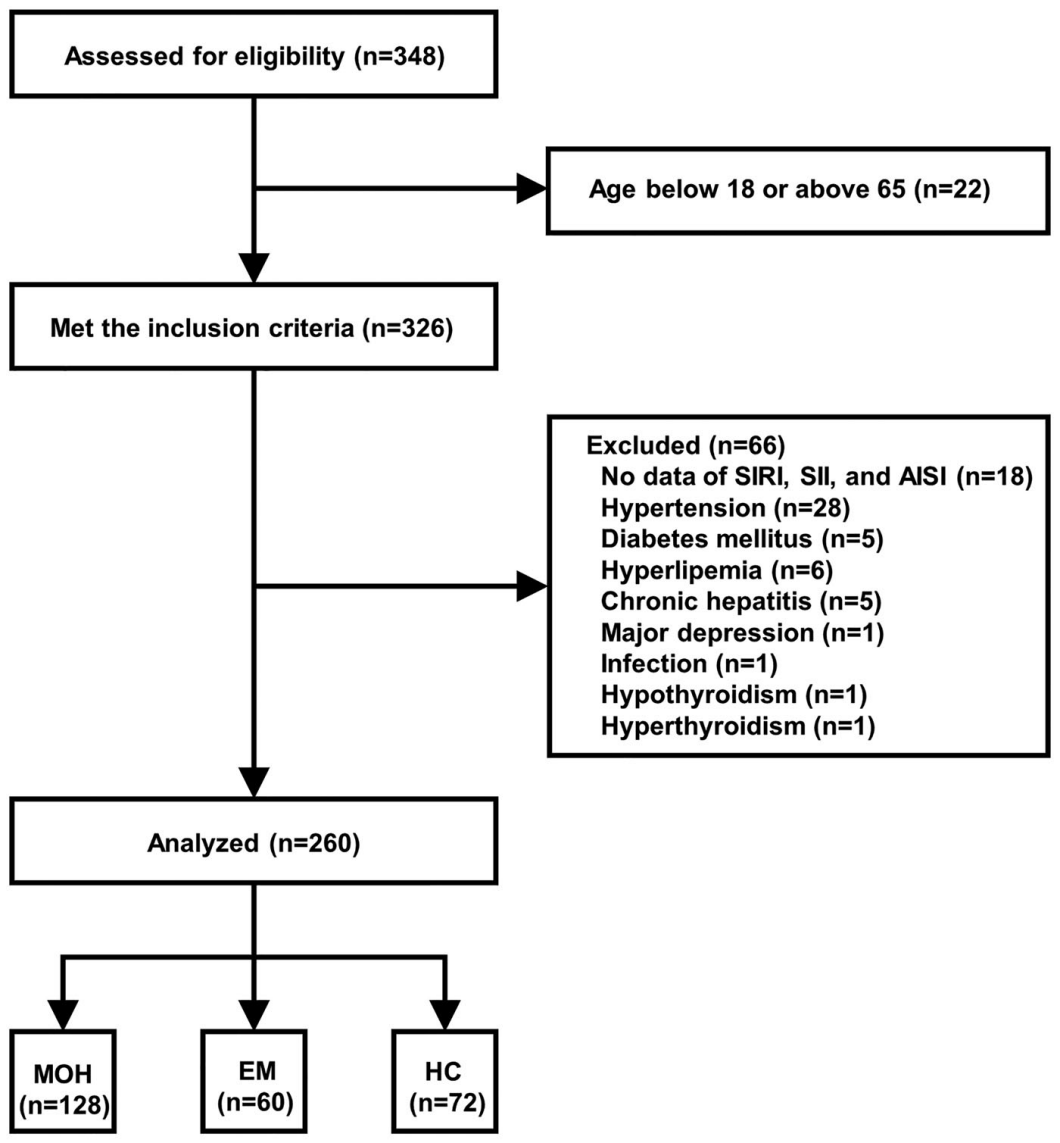
The flowchart of the study. **Abbreviations**: AISI, aggregate index of systemic inflammation; EM, episodic migraine; HC, healthy controls; MOH, medication overuse headache; SII, systemic inflammatory index; SIRI, systemic inflammation response index.

The flow diagram illustrating the participants included in the study is presented in Figure 1. Ultimately, a total of 260 participants were incorporated into the analysis, comprising 128 MOH patients, 60 EM controls, and 72 HC controls. The mean age of the MOH patients was 48.62 ± 9.06 years, while the EM controls had a mean age of 46.20 ± 8.10 years, and the HC controls had a mean age of 48.58 ± 6.42 years. The proportion of female participants was 72.7% in the MOH group, 71.7% in the EM group, and 73.6% in the HC group. The EM and HC groups were age‐ and sex‐matched with the MOH patients (shown in Table 1). No significant differences were observed between the MOH and EM groups in headache duration and intensity per month. However, the MOH group exhibited higher monthly headache days (24.49 ± 6.61 vs. 5.10 ± 2.68, p < 0.001) and days with acute medication (22.15 ± 7.69 vs. 2.98 ± 1.35, *p* < 0.001) compared to the EM group. Episodic migraine and TTH (63.3%) were the most common preexisting headaches in MOH patients, with a mean medication overuse duration of 4.51 ± 5.13 years, primarily involving compound analgesics. The main components of these analgesics were caffeine, aspirin, acetaminophen, aminopyrine, and phenacetin.

**TABLE 1:** Baseline characteristics and inflammatory indices of participants.

Characteristics	MOH (*n* = 128)	EM (*n* = 60)	HC (*n* = 72)	*p*
Age (year)	48.62 ± 9.06	46.20 ± 8.10	48.58 ± 6.42	0.104
Sex (female)	93 (72.7%)	43 (71.7%)	53 (73.6%)	0.969
Duration of headache (year)	16.17 ± 10.75	12.88 ± 7.19	——	0.070
Headache days per month	24.49 ± 6.61	5.10 ± 2.68	——	**< 0.001** ^*^
Headache intensity per month	6.11 ± 1.29	6.27 ± 1.10	——	0.314
Days per month with acute medication	22.15 ± 7.69	2.98 ± 1.35	——	**< 0.001** ^*^
Preexisting headache diagnoses				
Chronic migraine	7 (5.5%)	——	——	
Episodic migraine and TTH	81 (63.3%)	——	——	
Chronic TTH	40 (31.3%)	——	——	
Duration of medication overuse (year)	4.51 ± 5.13	——	——	
Type of medication overuse				
Simple analgesics	11 (8.6%)	——	——	
Triptans	2 (1.6%)	——		
Compound analgesics	115 (89.8%)	——	——	
Platelet	210.98 ± 63.05	191.97 ± 54.56	198.00 ± 59.58	0.095
WBC	6.10 ± 1.61^a^	5.83 ± 1.47	5.33 ± 1.25	**0.008** ^*^
Monocytes	0.40 ± 0.16^a^	0.37 ± 0.14	0.34 ± 0.11	**0.014** ^*^
Lymphocytes	1.87 ± 0.55	1.70 ± 0.49	1.71 ± 0.48	**0.035** ^*^
Neutrophils	3.67 ± 1.32^a^	3.57 ± 1.24	3.13 ± 0.96	**0.016** ^*^
NLR	2.13 ± 1.13	2.26 ± 1.02	1.95 ± 0.74	0.139
dNLR	0.86 ± 0.05	0.86 ± 0.07	0.86 ± 0.04	0.971
MLR	0.22 ± 0.09	0.23 ± 0.07	0.22 ± 0.12	0.175
NMLR	2.35 ± 1.17	2.48 ± 1.06	2.17 ± 0.83	0.118
PLR	120.19 ± 46.37	120.16 ± 46.09	124.54 ± 17.83	0.638
PWR	36.13 ± 12.53	34.36 ± 11.68	38.98 ± 13.88	0.102
SIRI	0.85 ± 0.56^a^	0.84 ± 0.46^a^	0.69 ± 0.45	**0.017** ^*^
SII	445.24 ± 278.12	430.77 ± 228.70	379.87 ± 170.80	0.314
AISI	182.44 ± 136.52^a^	164.24 ± 108.91	133.88 ± 100.17	**0.015** ^*^

*Note*: Values are presented as absolute numbers (percentages), or mean ± standard deviation. MOH, medication overuse headache; EM, episodic migraine; HC, healthy controls; TTH, tension‐type headache; WBC, white blood cells; NLR, neutrophil‐to‐lymphocyte ratio; dNLR, derived neutrophil‐to‐lymphocyte ratio; MLR, monocyte‐to‐lymphocyte ratio; NMLR, the ratio of (neutrophil count + monocyte count)/lymphocyte count; PLR, platelet‐to‐lymphocyte ratio; PWR, platelet‐to‐WBC ratio; SIRI, systemic inflammation response index; SII, systemic inflammatory index; AISI, aggregate index of systemic inflammation.

* Statistically significant at *p* < 0.05.

^a^ vs. HC group, adjusted *p* < 0.05.

After applying the Bonferroni adjustment, the MOH group exhibited significantly elevated values in WBC count (6.10 ± 1.61 vs. 5.33 ± 1.25, adjusted *p* = 0.006), monocyte count (0.40 ± 0.16 vs. 0.34 ± 0.11, adjusted *p* = 0.011), neutrophil count (3.67 ± 1.32 vs. 3.13 ± 0.96, adjusted *p* = 0.016), SIRI (0.85 ± 0.56 vs. 0.69 ± 0.45, adjusted *p* = 0.034), and AISI (182.44 ± 136.52 vs. 133.88 ± 100.17, adjusted *p* = 0.012) compared to the HC group. Additionally, the EM group demonstrated significantly higher SIRI levels (0.84 ± 0.46 vs. 0.69 ± 0.45, adjusted *p* = 0.042) than the HC group. While lymphocyte counts differed significantly among the three groups, no significant pairwise differences were observed after Bonferroni correction. No significant differences were found among the groups for platelet count, NLR, dNLR, MLR, NMLR, PLR, PWR, and SII (Table 1).

### 3.2 Comparison Among st‐MOH, Lt‐MOH, and HC

In this study, MOH patients were divided into short‐term (st‐MOH, ≤ 2 years) and long‐term (lt‐MOH, > 2 years) subgroups based on median medication overuse duration. No sex differences were found among the st‐MOH, lt‐MOH, and HC groups. The lt‐MOH subgroup was older (50.95 ± 8.13 vs. 46.76 ± 9.39, adjusted *p* = 0.031) and had longer headache duration (21.67 ± 11.52 vs. 11.75 ± 7.68, *p* < 0.001) than the st‐MOH subgroup, with no other clinical differences (Table 2).

**TABLE 2:** Comparison of clinical characteristics and inflammatory indices among st‐MOH, lt‐MOH, and HC groups.

Variables	st‐MOH (*n* = 71)	lt‐MOH (*n* = 57)	HC (*n* = 72)	*p*
Age (year)	46.76 ± 9.39^b^	50.95 ± 8.13	48.58 ± 6.42	**0.035** ^*^
Sex (female)	54 (76.1%)	39 (68.4%)	53 (73.6%)	0.620
Duration of headache (year)	11.75 ± 7.68	21.67 ± 11.52	——	**< 0.001** ^*^
Headache days per month	23.55 ± 7.02	25.67 ± 5.92	——	0.115
Headache intensity per month	5.99 ± 1.42	6.26 ± 1.09	——	0.219
Days per month with acute medication	21.07 ± 7.98	23.49 ± 7.15	——	0.077
Preexisting headache diagnoses				0.954
Chronic migraine	4 (5.6%)	3 (5.3%)	——	
Episodic migraine and TTH	44 (62.0%)	37 (64.9%)	——	
Chronic TTH	23 (32.4%)	17 (29.8%)	——	
Type of medication overuse				0.356
Simple analgesics	6 (8.5%)	5 (8.8%)	——	
Triptans	0 (0.0%)	2 (3.5%)	——	
Compound analgesics	65 (91.5%)	50 (87.7%)	——	
Platelet	209.93 ± 65.95	212.28 ± 59.79	198.00 ± 59.58	0.358
WBC	6.01 ± 1.57^a^	6.20 ± 1.67^a^	5.33 ± 1.25	**0.008** ^*^
Monocytes	0.40 ± 0.16^a^	0.40 ± 0.17	0.34 ± 0.11	**0.014** ^*^
Lymphocytes	1.90 ± 0.54	1.84 ± 0.57	1.71 ± 0.48	0.087
Neutrophils	3.54 ± 1.31	3.83 ± 1.33^a^	3.13 ± 0.96	**0.006** ^*^
NLR	2.03 ± 1.26	2.24 ± 0.93	1.95 ± 0.74	0.063
dNLR	0.86 ± 0.05	0.87 ± 0.04	0.86 ± 0.04	0.067
MLR	0.22 ± 0.09	0.23 ± 0.09	0.22 ± 0.12	0.268
NMLR	2.25 ± 1.30	2.47 ± 0.98	2.17 ± 0.83	0.067
PLR	115.61 ± 44.02	125.91 ± 48.93	124.54 ± 17.83	0.276
PWR	35.80 ± 11.47	36.53 ± 13.82	38.98 ± 13.88	0.280
SIRI	0.81 ±0.53	0.91 ±0.60^a^	0.69 ± 0.45	**0.013** ^*^
SII	426.64 ± 319.50	468.40 ± 216.40	379.87 ± 170.80	0.051
AISI	175.42 ± 139.95	191.19 ± 132.82^a^	133.88 ± 100.17	**0.006** ^*^

*Note*: Values are presented as absolute numbers (percentages), or mean ± standard deviation.

* Statistically significant at *p* < 0.05.

^a^ vs. HC group, adjusted p < 0.05.

^b^ vs. lt‐MOH group, adjusted *p* < 0.05.

**Abbreviations**: AISI, aggregate index of systemic inflammation; dNLR, derived neutrophil‐to‐lymphocyte ratio; HC, healthy controls; lt‐MOH, long‐term medication overuse headache; MLR, monocyte‐to‐lymphocyte ratio; NLR, neutrophil‐to‐lymphocyte ratio; NMLR, the ratio of (neutrophil count + monocyte count)/lymphocyte count; PLR, platelet‐to‐lymphocyte ratio; PWR, platelet‐to‐WBC ratio; SII, systemic inflammatory index; SIRI, systemic inflammation response index; st‐MOH, short‐term medication overuse headache; TTH, tension‐type headache; WBC, white blood cells.

Significant differences in WBC, monocyte, neutrophil counts, SIRI, and AISI levels were found among the groups (p < 0.05, Table 2). Post hoc analysis revealed that the st‐MOH group had higher WBC (6.01 ± 1.57 vs. 5.33 ± 1.25, adjusted *p* = 0.043) and monocyte counts (0.40 ± 0.16 vs. 0.34 ± 0.11, adjusted *p* = 0.024) compared to HC, while the lt‐MOH group had higher WBC (6.20 ± 1.67 vs. 5.33 ± 1.25, adjusted *p* = 0.014), neutrophil counts (3.83 ± 1.33 vs. 3.13 ± 0.96, adjusted *p* = 0.004), SIRI (0.91 ± 0.60 vs. 0.69 ± 0.45, adjusted *p* = 0.010), and AISI (191.19 ± 132.82 vs. 133.88 ± 100.17, adjusted *p* = 0.005). No significant differences in these parameters were found between the st‐MOH and lt‐MOH groups.

### 3.3 Correlation Analysis Between Inflammatory Biomarkers and Clinical Phenotypes

All inflammatory indices, except for dNLR and PWR, showed a positive correlation with the duration of headache in MOH patients (*p* < 0.05, shown in Table 3). Furthermore, SIRI and AISI were positively correlated with the days per month with acute medication (*r* = 0.180, *p* = 0.042; *r* = 0.200, *p* = 0.024, respectively). However, none of the inflammatory biomarkers were significantly correlated with the headache days or intensity per month or the duration of medication overuse in MOH patients.

**TABLE 3:** Correlations between inflammatory biomarkers and clinical phenotypes.

Biomarkers	Duration of headache		Days per month with acute medication	
	*r*	*p*	*r*	*p*
NLR	0.186	0.036^*^	0.135	0.128
dNLR	0.016	0.859	0.032	0.723
MLR	0.221	0.012^*^	0.088	0.322
NMLR	0.198	0.025^*^	0.136	0.125
PLR	0.199	0.024^*^	0.068	0.444
PWR	0.043	0.626	−0.003	0.974
SIRI	0.222	0.012^*^	0.180	0.042^*^
SII	0.217	0.014^*^	0.160	0.071
AISI	0.225	0.011^*^	0.200	0.024^*^

**Abbreviations**: AISI, aggregate index of systemic inflammation; dNLR, derived neutrophil‐to‐lymphocyte ratio; MLR, monocyte‐to‐lymphocyte ratio; NLR, neutrophil‐to‐lymphocyte ratio; NMLR, the ratio of (neutrophil count + monocyte count)/lymphocyte count; PLR, platelet‐to‐lymphocyte ratio; PWR, platelet‐to‐WBC ratio; SII, systemic inflammatory index; SIRI, systemic inflammation response index.

### 3.4 Performance of Systemic Inflammatory Biomarkers for the Identification of MOH

**FIGURE 2:**
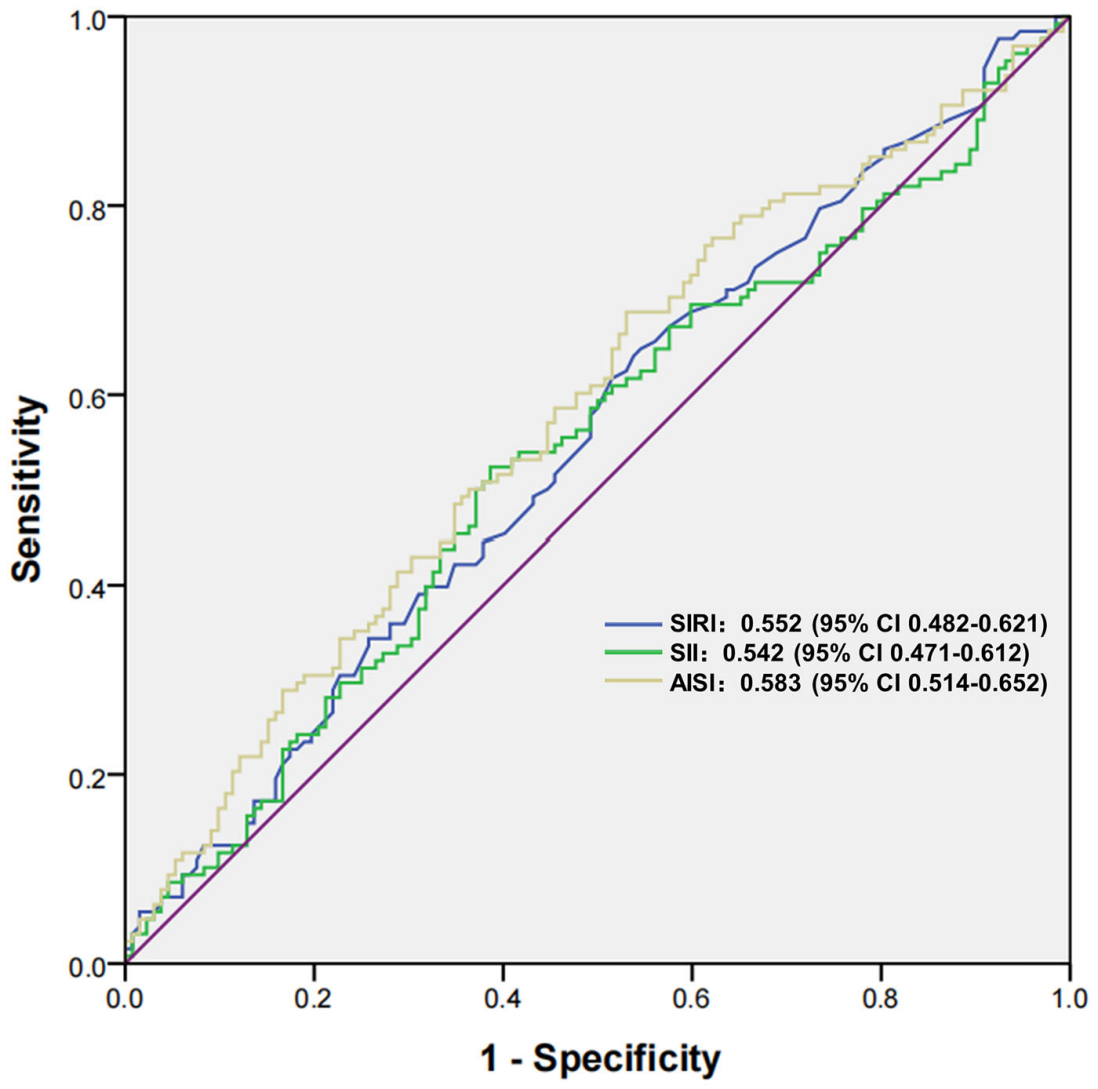
Comparison of the diagnostic values of SIRI, SII, and AISI for MOH. **Abbreviations**: AISI, aggregate index of systemic inflammation; MOH, medication overuse headache; SII, systemic inflammatory index; SIRI, systemic inflammation response index.

The diagnostic performances of SIRI, SII, and AISI are illustrated in Figure 2. The AUC for AISI in identifying MOH was 0.583 (95% CI 0.514–0.652, *p* = 0.020), with a cut‐off value of 104.95. The SII identified MOH with an AUC of 0.542 (95% CI 0.471–0.612, *p* = 0.244) and a cut‐off at 406.24, while the SIRI identified MOH with an AUC of 0.552 (95% CI 0.482–0.621, *p* = 0.151) and a threshold value of 0.59. In comparison, AISI demonstrated superior identification capability for MOH.

### 3.5 Association Between Systemic Inflammatory Biomarkers and MOH

Participants were categorized into low and high groups for AISI, SII, and SIRI based on optimal cut‐off values. In the univariate logistic regression analysis, elevated levels of SII (≥ 406.24) and AISI (≥ 104.95) significantly correlated with MOH (Table 4). After adjusting for age and sex, higher levels of SII (OR 1.76, 95% CI 1.08–2.89, *p* = 0.025) and AISI (OR 2.00, 95% CI 1.20–3.34, *p* = 0.008) were independently associated with MOH.

**TABLE 4:** Logistic regression examining the association of SII, SIRI, and AISI with the risk of MOH.

Variables	Cut‐off point	Crude model		Adjusted model	
		OR (95%CI)	*p*	OR (95%CI)	*p*
SIRI	< 0.59	Reference	0.093	Reference	0.081
	≥ 0.59	1.53 (0.93‐2.52)		1.56 (0.95‐2.56)	
SII	< 406.24	Reference	**0.027** ^*^	Reference	**0.025** ^*^
	≥ 406.24	1.74 (1.07–2.86)		1.76 (1.08–2.89)	
AISI	< 104.95	Reference	**0.010** ^*^	Reference	**0.008** ^*^
	≥ 104.95	1.95 (1.17–3.23)		2.00 (1.20–3.34)	

*Note*: Crude model adjusted no variables. Adjusted model adjusted age and sex.

* Statistically significant at *p* < 0.05.

**Abbreviations**: AISI, aggregate index of systemic inflammation; SII, systemic inflammatory index; SIRI, systemic inflammation response index.

## 4 Discussion

This study investigated peripheral blood count‐derived inflammatory indices as biomarkers of systemic inflammation in headache disorders, focusing on differences between MOH patients and controls and their correlation with MOH clinical phenotypes. Compared to HC, AISI levels were higher in MOH patients, while SIRI levels were elevated in both MOH and EM patients. No significant differences were observed in these indices between the st‐MOH and lt‐MOH groups. AISI and SIRI correlated positively with days per month with acute medication, and several indices (NLR, MLR, NMLR, PLR, SIRI, SII, and AISI) correlated with duration of headache in MOH patients. AISI had better diagnostic value for MOH than SII and SIRI, with elevated SII and AISI levels independently associated with MOH.

AISI, SIRI, and SII are key indicators of systemic inflammation, reflecting the balance between adaptive (lymphocytes) and innate (platelets, neutrophils, monocytes) immune cells (Hamad et al. 2022; Hu et al. 2014; Paliogiannis et al. 2020; Vázquez‐Mojena et al. 2023). This study found that AISI and SIRI levels, but not SII, were significantly higher in MOH patients and the lt‐MOH subgroup compared to HC. This suggested that elevated SIRI and AISI levels might result from chronic innate immune activation, mainly due to increased monocytes. Monocytes are crucial in both innate and adaptive immunity (Ginhoux et al. 2020; Guilliams et al. 2018), secreting pro‐inflammatory mediators like tumor necrosis factor and interleukins (Gane et al. 2016). They, along with neutrophils, initiate the innate immune response, with platelet‐neutrophil interactions playing a vital role (Koupenova et al. 2018; Margraf and Zarbock 2019). Our findings are consistent with previous research suggesting the neuroinflammatory immunity's role in MOH (Gong et al. 2023; Wang et al. 2023). We found EM patients had higher SIRI levels than HC, unlike a recent study (Kömürcü et al. 2025). This difference might be due to age, sample size, headache frequency, and other factors.

In MOH patients, AISI and SIRI levels correlated with duration of headache and days per month with acute medication, while SII correlated with duration of headache. High SII and AISI levels were significantly associated with MOH presence. MOH diagnosis depends solely on official clinical criteria, with some patients having poor prognoses (Grodzka et al. 2025; Koonalintip et al. 2025). Compared to cytokines (Vuralli et al. 2024; Vuralli, Ceren Akgor, Gok Dagidir, et al. 2024), SII, AISI, and SIRI may be more cost‐effective biomarkers for diagnosing and predicting MOH outcomes. Our findings suggested that peripheral inflammation may worsen headache symptoms and increase acute medication use in MOH patients, or that chronic headaches might trigger peripheral inflammation. Previous studies indicated that increased peripheral immune cells (lymphocytes) could be a non‐specific response to chronic pain, potentially leading to analgesic overuse (Forcelini et al. 2011). However, due to the cross‐sectional design of these studies, causation is unclear, highlighting the need for longitudinal research.

This study's systemic inflammation indices (SII, SIRI, and AISI) provided a comprehensive view of inflammation from routine blood tests, while earlier research identified specific inflammatory markers like IL‐6 and IL‐17 in MOH, pointing to cytokine‐mediated inflammation (Dağıdır et al. 2023; Vuralli et al. 2024). The performance differences between these markers were noteworthy: IL‐6 and IL‐17 may indicate specific acute inflammation, while our composite indices might represent a broader, ongoing systemic state not exclusive to MOH. Although SII and AISI levels were independently associated with MOH, their low diagnostic specificity (AUC < 0.6) limited their standalone clinical use, similar to lymphocyte count and neutrophil/lymphocyte ratio in distinguishing MOH from EM and HC (Grodzka et al. 2025). This may be due to MOH's variability from different primary headaches or overused medications, non‐specific inflammation, central mechanisms (e.g., reward circuit remodeling) overshadowing peripheral ones, and lifestyle or emotional factors. Despite promising MO/MOH prediction models in migraine patients (Aramruang et al. 2024), the field is still developing. Future studies should integrate biomarker data, particularly blood (such as IL‐6, IL‐17, SII, AISI, etc.) and imaging biomarkers, with clinical features to better understand their roles and enhance MOH diagnosis through a multimodal approach. Recently, the European Headache Federation suggested integrating community pharmacists into headache management services (BaniHani et al. 2025).

This study found no significant differences in NLR, dNLR, MLR, NMLR, PLR, and PWR among MOH, EM patients, and HC, or between st‐MOH and lt‐MOH subgroups. However, NLR, MLR, NMLR, and PLR were positively correlated with headache duration in MOH patients. These results partially contradict earlier findings that MOH patients had higher NLR levels than EM patients and HC, and that lower NLR was associated with fewer headache days/month (Carlsen et al. 2023). Previous studies on NLR, PLR, and MLR levels between EM patients and HC have been inconsistent, showing varied results (Kömürcü et al. 2025; Lee et al. 2022; Sarıcam 2021; Wu et al. 2024; Yazar et al. 2020). NLR was related to headache intensity, and MLR to headache frequency in migraine patients (Wu et al. 2024). These discrepancies may stem from differences in sample size, migraine subtypes, and blood sample timing (e.g. during interictal periods versus headache attacks), which could affect inflammatory responses.

The link between peripheral inflammation and MOH is unclear but may involve the brain‐gut axis and blood‐brain barrier issues. Overuse of nonsteroidal anti‐inflammatory drugs (NSAIDs) can damage the intestinal barrier, allowing lipopolysaccharides to enter the bloodstream, triggering inflammation and cytokine release, which worsens the barrier and may cause systemic inflammation in MOH (Dağıdır et al. 2023; Vuralli et al. 2024; Vuralli, Ceren Akgor, Gok Dagidir, et al. 2024). Pro‐inflammatory cytokines like IL‐6 and IL‐1β contributed to headaches by affecting the trigeminovascular system (Dağıdır et al. 2023). Additionally, peripheral inflammation (e.g., lipopolysaccharide exposure) can compromise the blood‐brain barrier, allowing inflammatory molecules (e.g., IL‐6) and pathogens to reach the central nervous system, worsening neuroinflammation (Zhang et al. 2023). Neuroinflammation may influence peripheral inflammation through neuroimmune pathways (e.g., lymphatic system involvement) or neurotransmitter changes, such as calcitonin gene‐related peptide signaling (Passaro et al. 2021; Vázquez‐Mojena et al. 2023). In MOH, chronic medication abuse might exacerbate this, causing persistent barrier damage and systemic inflammation cycles.

This study possesses several limitations that warrant consideration. First, we did not measure key signaling molecules like interleukins, despite previous findings of higher IL‐6 in female chronic migraine patients with MOH (Vuralli, Ceren Akgor, Gok Dagidir, et al. 2024). Second, the absence of chronic migraine patients without MOH limited conclusions on whether inflammation was specific to MOH or a general chronic headache trait. It's unclear if low‐grade systemic inflammation stems from chronic headaches or overuse of acute medications. Notably, up to 90% of MOH patients had EM without medication overuse before MOH, and about 70% returned to EM without medication overuse after treatment (Carlsen et al. 2023), supporting the use of EM controls. The significant differences in headache frequency and medication exposure between MOH and EM groups could introduce confounding factors. Future research should include chronic migraine patients to better identify the source of inflammatory changes. Third, although we controlled for age and sex and excluded participants with other chronic conditions or on prophylactic medications, other confounding factors cannot be completely ruled out. Fourth, previous studies suggested MOH patients overusing triptans may focus on central sensitization, while NSAID overuse exhibited a systemic inflammatory profile (Takahashi et al. 2021; Vuralli et al. 2024; Vuralli, Ceren Akgor, Gok Dagidir, et al. 2024). We did not stratify MOH patients by specific analgesic types due to the relatively small sample size and mixed analgesic use, which may affect the observed associations. Thus, our findings should be interpreted cautiously, especially given the relatively small sample size and cross‐sectional nature of the analysis. Future studies should involve larger, multi‐center longitudinal studies to determine if inflammation drives MOH or results from chronic headaches or medication overuse.

## 5 Conclusion

This study found SII, SIRI, and AISI to be useful biomarkers of peripheral inflammation in MOH, linking clinical phenotypes like headache duration and days per month with acute medication to chronic immune activation. Elevated SII and AISI levels were significantly associated with MOH, suggesting these biomarkers could improve understanding and diagnosis of MOH.

## Author Contributions

**Changling Li**: conceptualization, data curation, formal analysis, investigation, validation, visualization, writing–original draft, writing–review and editing. **Peiqi He**: conceptualization, data curation, formal analysis, investigation, validation, visualization, writing–original draft, writing–review and editing. **Mengmeng Ma**: conceptualization, investigation, methodology, project administration, supervision, writing–review and editing. **Yanbo Li**: conceptualization, funding acquisition, methodology, supervision, writing–review and editing. **Jinghuan Fang**: conceptualization, methodology, supervision, writing–review and editing. **Qian Liu**: formal analysis, methodology, visualization, supervision, writing–review and editing. **Yang Zhang**: formal analysis, methodology, validation, supervision, writing–review and editing. **Xin Jiang**: data curation, investigation, writing–review and editing. **Shiqin Li**: data curation, writing–review and editing. **Hui Lang**: data curation, writing–review and editing. **Ning Chen**: conceptualization, formal analysis, resources, project administration, supervision, writing–review and editing. **Li He**: conceptualization, funding acquisition, resources, project administration, supervision, writing–review and editing.

## Funding

This project was supported by the Postdoctoral Research Fund of West China Hospital, Sichuan University (grant number: 2024HXBH069), the National Key Research and Development Program of China (grant number: 2023YFC2508702), the Natural Science Foundation of China (grant number: 82301404), the Natural Science Foundation of Sichuan Province (grant number: 2023NSFSC1562), and the Sichuan Science and Technology Program (grant number: 2022YFS0139).

## Ethics Statement

This cross‐sectional study was approved by the Ethics Committee of West China Hospital, Sichuan University (No. 2020‐666).

Consent

All participants provided verbal informed consent before inclusion.

## Conflicts of Interest

The authors declare no conflicts of interest.

## Data Availability

The datasets of the current study could be obtained from the corresponding author upon appropriate request.
